# Expression of a Salt-Tolerant Pseudolysin in Yeast for Efficient Protein Hydrolysis under High-Salt Conditions

**DOI:** 10.3390/biom13010083

**Published:** 2022-12-30

**Authors:** Xiufang Liu, Qian Lu, Han Xiao, Yunzi Feng, Guowan Su, Mouming Zhao, Mingtao Huang

**Affiliations:** 1 School of Food Science and Engineering, South China University of Technology, Guangzhou 510641, China; 2 Guangdong Food Green Processing and Nutrition Regulation Technologies Research Center, Guangzhou 510650, China; 3 Guangdong Haitian Innovation Tech Co., Ltd., Foshan 528000, China

**Keywords:** recombinant protease, *Saccharomyces cerevisiae*, salinity resistance, enzyme properties, soy protein isolate hydrolyzation

## Abstract

Protease biocatalysis in a high-salt environment is very attractive for applications in the detergent industry, the production of diagnostic kits, and traditional food fermentation. However, high-salt conditions can reduce protease activity or even inactivate enzymes. Herein, in order to explore new protease sources, we expressed a salt-tolerant pseudolysin of *Pseudomonas aeruginosa* SWJSS3 isolated from deep-sea mud in *Saccharomyces cerevisiae*. After optimizing the concentration of ion cofactors in yeast peptone dextrose (YPD) medium, the proteolytic activity in the supernatant was 2.41 times more than that in the control group when supplemented with 5 mM CaCl_2_ and 0.4 mM ZnCl_2_. The extracellular proteolytic activity of pseudolysin reached 258.95 U/mL with optimized expression cassettes. In addition, the *S. cerevisiae* expression system increased the salt tolerance of pseudolysin to sodium chloride (NaCl)and sodium dodecyl sulfate (SDS) and the recombinant pseudolysin retained 15.19% activity when stored in 3 M NaCl for 7 days. The recombinant pseudolysin was able to efficiently degrade the β-conglycinin from low-denatured soy protein isolates and glycinin from high-denatured soy protein isolates under high temperatures (60 °C) and high-salt (3 M NaCl) conditions. Our study provides a salt-tolerant recombinant protease with promising applications in protein hydrolysis under high-salt conditions.

## 1. Introduction

Proteases have a diverse range of eco-friendly industrial applications, accounting for approximately 40% of enzyme market sales [1]. An attractive market for protease is its catalysis in a high-salt environment, generally used as the main ingredient in detergents [2], additives in diagnostic kits [3], and protein hydrolysis [4] in high-salt food fermentation. However, biocatalytic reactions are greatly limited under hypersaline conditions due to reduced enzyme activity and the poor salt tolerance of protease. So far, only a few studies have focused on improving the salt tolerance of proteases by limiting the access of salt ions to the active site [3,5], increasing the negative potential [6], and improving the affinity of the primary Ca^2+^ binding site [7]. Therefore, it is necessary to discover and develop salt-tolerant proteases to meet industrial needs in a sustainable way.

In our previous study, a salt-tolerant protease was separated from *Pseudomonas aeruginosa* SWJSS3. The strain *P. aeruginosa* SWJSS3 was isolated from deep-sea mud through enrichment with high-salt cultivation [8]. This protease maintains 40.70% activity when mixed with 2.57 M NaCl for 3 h. The protease was later identified as pseudolysin, a member of the thermolysin family. Besides salt tolerance, pseudolysin has also been noted for its keratinolytic activity [9], depilating capabilities [10,11], deproteinization of shrimp waste [12], and organic solvent stability [13,14]. However, *P. aeruginosa* is an opportunistic pathogen and can cause life-threatening infections, particularly in immune-deficient patients [15]. The application of products derived from *P. aeruginosa* may be limited. Therefore, the heterologous expression of the protease is considered to be environmentally friendly and safe. In the past, pseudolysin has been successfully expressed in *Escherichia coli* as an intracellular format [14,16,17]. However, the intracellular expression of pseudolysin encountered inactive insoluble inclusion (inclusion bodies) problems and required additional refolding steps, which added challenges to large-scale purification [18,19,20]. Researchers have also expressed pseudolysin in *Pichia pastoris* using methanol induction, thus secreting mature pseudolysin [21,22,23,24]. *Saccharomyces cerevisiae* has “generally regarded as safe” (GRAS) status and is widely used for producing recombinant proteins [25]. Moreover, *S. cerevisiae,* as an eukaryotic model organism, is able to implement complex post-translational modification and secrete appropriately folded and functional proteins [26]. Therefore, the heterologous expression of pseudolysin by *S. cerevisiae* could be an alternative way to expand expression systems used in the production of pseudolysin.

In this work, we used *S. cerevisiae* as a host to secrete the salt-tolerant protease from *P. aeruginosa* SWJSS3 and increase proteolytic activity by optimizing the concentration of ion cofactors in the medium. Recombinant pseudolysin produced by *S. cerevisiae* showed enhanced salt tolerance toward NaCl and SDS. Finally, we successfully applied the recombinant pseudolysin by degrading two soy protein isolates under a high-salt environment.

## 2. Materials and Methods

### 2.1. Plasmids and Strains

The plasmids and strains used in this study are listed in Table 1 and Table 2. The polymerase chain reaction (PCR) primers are listed in Table S1. The *lasB* gene encoding pseudolysin was amplified from the genome of *P. aeruginosa* SWJSS3 and sequenced by Sangon Biotech Co., Ltd. (Shanghai, China). The sequence of the wildtype pseudolysin from the standard *P. aeruginosa* PA01 strain was obtained from the Uniprot (Entry: P14756). The *lasB* gene from *P. aeruginosa* SWJSS3 was codon-optimized and -synthesized by GenScript (Nanjing, China).

The *Escherichia coli* DH5α was used for plasmid construction and propagation. In order to construct protease expression vectors, the multicopy plasmid CPOTud was linearized by digesting with restriction enzymes Kpn I and Nhe I. DNA fragment amplification was performed using the Phanta^®^ Max Super-Fidelity DNA Polymerase (Cat# P525-02, Vazyme, Nanjing, China) and was inserted into the cloning site of plasmid backbones by ligation via T4 DNA ligase (Thermo Scientific, Waltham, USA). Then, ligation products were transformed into *Escherichia coli* DH5α with standard molecular biology techniques. After verification, plasmids were extracted from *E. coli* and transformed into the *S. cerevisiae* strains CEN.PK 530-1C and B184M using the LiAc/SS-DNA/PEG method [27].

**Table 1:** Plasmids used in this study.

Plasmids	Description	Source
CPOTud	2 μm, AmpR, TPI1p, TPI1t, POT1 gene from *S. pombe* as a selection marker.	[28]
pAlphaAmyCPOT	CPOTud with amylase gene driven by α-factor prepro leader	[28]
plasB1	CPOTud- (native signal peptide + mature domain of mutant pseudolysin)	This study
plasB2	CPOTud- (native signal peptide + propeptide + mature domain of mutant pseudolysin)	This study
plasB3	CPOTud- (α-factor pre leader + mature domain of mutant pseudolysin)	This study
plasB4	CPOTud- (α-factor pre leader + propeptide + mature domain of mutant pseudolysin)	This study
plasB5	CPOTud- (α-factor prepro leader + mature domain of mutant pseudolysin)	This study
plasB6	CPOTud- (α-factor prepro leader + propeptide + mature domain of mutant pseudolysin)	This study
plasB7	CPOTud- (PIR1 pre leader + propeptide + mature domain of mutant pseudolysin)	This study
plasB8	CPOTud- (PIR1 prepro leader + propeptide + mature domain of mutant pseudolysin)	This study
PlasB9	CPOTud- (SCW10 pre leader + propeptide + mature domain of mutant pseudolysin)	This study
PlasB10	CPOTud- (SCW10 prepro leader + propeptide + mature domain of mutant pseudolysin)	This study

**Table 2:** Strains used in this study.

Strains	Description	Source
*P. aeruginosa* SWJSS3	Strain-secreting mutant pseudolysin	[8]
*E. coli* DH5α	F^-^ Φ80*lac*ZΔM15 Δ(*lac*ZYA-*arg*F) U169 *rec*A1 *end*A1 *hsd*R17(r_k_^-^, m_k_^+^) *pho*A *sup*E44 *thi*-1 *gyr*A96 *rel*A1λ^-^	AngYuBio Co., Ltd.
*S. cerevisiae* CEN.PK 530-1C	MATa tpi1(41-707)::loxP-KanMX4-loxP	[29]
B184M	The UV mutant strain obtained from CEN.PK 530-1C	[30]
K0	CEN.PK 530-1C/CPOTud	This study
K_lasB1	CEN.PK 530-1C/plasB1	This study
K_lasB2	CEN.PK 530-1C/plasB2	This study
K_lasB3	CEN.PK 530-1C/plasB3	This study
K_lasB4	CEN.PK 530-1C/plasB4	This study
K_lasB5	CEN.PK 530-1C/plasB5	This study
K_lasB6	CEN.PK 530-1C/plasB6	This study
K_lasB7	CEN.PK 530-1C/plasB7	This study
K_lasB8	CEN.PK 530-1C/plasB8	This study
K_lasB9	CEN.PK 530-1C/plasB9	This study
K_lasB10	CEN.PK 530-1C/plasB10	This study
B0	B184M /CPOTud	This study
B_lasB1	B184M/plasB1	This study
B_lasB2	B184M/plasB2	This study
B_lasB3	B184M/plasB3	This study
B_lasB4	B184M/plasB4	This study
B_lasB5	B184M/plasB5	This study
B_lasB6	B184M/plasB6	This study
B_lasB7	B184M/plasB7	This study
B_lasB8	B184M/plasB8	This study
B_lasB9	B184M/plasB9	This study
B_lasB10	B184M/plasB10	This study

### 2.2. Media and Cultivations

*E. coli* DH5α with a transformed plasmid was grown in LB medium, consisting of 5 g/L yeast extract, 10 g/L tryptone, and 10 g/L NaCl with a supplement of 100 mg/L ampicillin for plasmid propagation. For recombinant pseudolysin production in tubes, *S. cerevisiae* with an expression plasmid was cultured in 2.5 mL of YPD media (10 g/L yeast extract, 20 g/L peptone, and 20 g/L dextrose) in the presence of 5 mM CaCl_2_ plus 0.4 mM ZnCl_2_ for 96 h at 30 °C and 200 rpm. For recombinant pseudolysin purification, yeast strains were grown overnight in YPD medium at 30 °C and then transferred into 200 mL of fresh YPD medium with a supplement of 5 mM CaCl_2_ plus 0.4 mM ZnCl_2_ to an OD_600 nm_ of 0.12 and cultivated for 96 h. To secret mutant pseudolysin, a single *P. aeruginosa* SWJSS3 colony was incubated in 10 mL of LB broth overnight at 37 °C, combined with shaking at 200 rpm for seed culture. Subsequently, 1 mL of the seed culture was transferred into a 100 mL fermentation medium at 30 °C and cultivated for 36 h. The fermentation medium consisted of 4 g/L glucose, 16 g/L yeast extract, 8 g/L glycerol, 0.2 g/L dipotassium hydrogen phosphate, 0.5 g/L Tween-80, and 10 g/L sodium chloride (pH7.2 ± 0.1).

### 2.3. Pseudolysin Activity Assay

The pseudolysin activity measurement was carried out using the method described by Zhu [31] with slight modifications. After cultivation at 30 °C for 96 h, the supernatant of yeast culture was collected for the detection of protease activity by centrifugation at 12,000 rpm for 2 min and then was appropriately diluted with 50 mM Tris-HCl (pH = 7.5). Two hundred microliters of supernatant dilution was added to 200 μL of 20 g/L casein solution. After appropriate incubation for 10 min at 40 °C and 400 μL of 10% trichloroacetic acid was added to the mixture to terminate the reaction. The precipitation was removed by centrifugation at 14,000 rpm for 2 min, and the absorbance of the supernatant was determined at 275 nm with a UV–vis spectrophotometer (T2600, Shanghai Yoke Instrument. Co., Ltd., Shanghai, China). One unit of pseudolysin activity was defined as the number of enzymes needed to catalyze the substrate to produce 1 μg of tyrosine.

For the intracellular protease activity quantitation, 500 μL of *S. cerevisiae* fermentation broth was centrifuged at 12,000 rpm for 2 min. The cell pellet was washed twice with 0.01 M PBS, resuspended in 500 μL PBS, and transferred into a 2 mL tube with 0.7 g of 0.5 mm glass beads (Biospec Products Inc., Bartlesville, USA). The cell suspension was disrupted by a homogenizer (Bioprep-24R, Hangzhou Allsheng Instrument Co., Ltd., Hangzhou, China) for 2 min. After centrifugation, the supernatant was used to measure intracellular protease activity.

### 2.4. Protein Purification

Regarding the mutant pseudolysin from *P. aeruginosa* SWJSS3, after cultivation at 30 °C for 36 h, 100 mL of supernatant was harvested by centrifugation for 10 min at 12,000 rpm to remove the cell pellet and was concentrated with an Amicon^®^ Ultra-15 centrifugal filter (30 kDa molecular weight cut-off). Then, 2 mL of the above-collected protein solution was loaded into a HiTrap^TM^ 1 mL Capto^TM^ Q chromatography column (Cytiva, Marlborough, USA) pre-equilibrated with 50 mM Tris-HCl and a pH 8.0, at a flow rate of 1 mL/min. The column was washed with a linear gradient NaCl (0–500 mM) in 50 mM Tris-HCl, pH = 8.0, on an ÄKTA pure 150 chromatography system (Cytiva, Marlborough, USA). The eluates were collected automatically using a 1 mL/tube, where the buffer was replaced by 50 mM Tris-HCl (pH = 8.0) through ultrafiltration, and were stored at −80 °C.

For the recombinant pseudolysin, the fermentation broth was centrifuged, and 300 mL of supernatant was concentrated and buffer-displaced through an Amicon^®^ Ultra-15 centrifugal filter (30 kDa molecular weight cut-off) for discarding unwanted proteins from the YPD medium. Next, 5 mL of concentrated recombinant pseudolysin was applied to a Hitrap^TM^ 1 mL Capto^TM^ DEAE chromatography column (Cytiva, Marlborough, USA) with an equilibration of 50 mM Tris-HCl, pH = 8.0, at 1 mL/min. Recombinant pseudolysin was purified via the collection of flow-through fractions when sample loading and protein impurities combined with resin were removed with washing buffer (50 mM Tris-HCl, 1 M NaCl, pH = 8.0).

After purification, the protein concentration was determined using a BCA protein assay kit with bovine serum albumin as the reference molecule (Sangon Biotech Co., Ltd., Shanghai, China), and protease activity was also measured. Then, collected fractions were analyzed by sodium dodecyl sulfate polyacrylamide gel electrophoresis (SDS-PAGE) to show the major composition within purified samples. The protein sample was mixed with 5× loading buffer, fully denatured at 100 °C for 10 min, and loaded into 4–12% SurePAGE precast gel (Nanjing GenScript Co., Ltd., Nanjing, China). After electrophoresis, the gel was stained with Coomassie Brilliant Blue and imaged using a ChemiDoc™ XRS+ System (Bio-Rad, Hercules, USA).

### 2.5. Western Blot of Recombinant Pseudolysin

Ten microliters of fermentation supernatant were analyzed by SDS-PAGE as described previously and transferred to polyvinylidene difluoride (PVDF) membrane at 200 mA for 2 h. Then, the PVDF membrane was blocked with QuickBlock™ Blocking Buffer (Shanghai Beyotime Biotechnology Co., Ltd., Shanghai, China) for 30 min and incubated with THE™ His Tag Antibody (Nanjing GenScript Co., Ltd., Nanjing, China) for 2 h at 25 °C. The secondary antibody reaction was carried out using an HRP-labeled Goat Anti-Mouse IgG(H+L) (Shanghai Beyotime Biotechnology Co., Ltd., Shanghai, China) incubation for 2 h. The membrane was washed three times for 10 min with Tris-buffered saline with 0.1% Tween (TBST) every two steps. Finally, the protein bands were detected via Pierce™ ECL Western Blotting Substrate (Thermo Scientific, Waltham, USA) and visualized using the ChemiDoc™ XRS+ System (Bio-Rad, Hercules, USA).

### 2.6. Enzyme Kinetics and General Characteristics

In order to determine enzyme kinetics, two hundred microliters of purified SC-lasB and PA-lasB were added to 200 μL of casein solution at various concentrations ranging from 1.25 to 20 g/L [32]. The reaction mixture was incubated at 60 °C over different times (2.5–12.5 min) and was terminated by adding 400 μL of 10% trichloroacetic acid. Then, the released tyrosine was quantified. The initial reaction velocity (V_0_) was calculated using the linear slope between the tyrosine concentration and increasing reaction time at each casein concentration. Then, the V_0_ was plotted over casein concentration and fitted to the nonlinear regression of the Michaelis–Menten equation with GraphPad Prism 9. Vmax and Kcat were obtained based on the fitness result of the Michaelis–Menten equation, and Km was calculated by dividing Kcat by the mass concentration of purified SC-lasB and PA-lasB.

The salt tolerance of pseudolysins was assessed by mixing enzyme (100 μg/mL) with NaCl to a final concentration from 0.5 M to 4 M and storing it at 4 °C for 1 h. Long-term salt tolerance was evaluated by incubation with 2 M and 3 M NaCl for 7 days. Similarly, the SDS resistance was examined through incubation at 0.05% to 0.10% SDS for 1 h. To determine the optimum temperature, the enzyme activity of SC-lasB and PA-lasB was measured in a range of 30 °C to 80 °C for a 10 min reaction. The value of the highest enzyme activity was defined as 100%. The thermal stability was evaluated by residual activity after incubation at 50 °C, 60 °C, and 70 °C for 0, 20, 40, and 60 min, respectively. The enzyme activity of untreated samples (0 min) was defined as 100%.

### 2.7. Soy Protein Isolate Hydrolysis

Low-denatured soy protein isolates (LD-SPI) (Hainan Hongke Biotechnology Co., Ltd., Sanya, China) and high-denatured soy protein isolates (HD-SPI) (Shandong Jiahua Biotechnology Co., Ltd., Liaocheng, China) were used for enzymatic hydrolysis test. SPI was dispersed in 3 M NaCl to attain a final protein concentration of 10 mg/mL, which was gently stirred for 2 h, then stored at 4 °C overnight to achieve adequate hydration [33]. The enzymatic hydrolysis of SPI was carried out in conditions of 60 °C and pH = 7.0 for the two pseudolysins or 50 °C and pH = 8.0 for porcine pancreatic elastase (Shanghai Macklin Biochemical Co., Ltd., Shanghai, China). Subsequently, the reaction was terminated at 90 °C for 20 min to inactivate the enzyme, and it was then centrifuged at 14,000 rpm for 5 min. The supernatant was carefully collected and used for SDS-PAGE analysis.

## 3. Results and Discussion

### 3.1. Bioinformatic Analysis of the lasB Gene from P. aeruginosa SWJSS3

The entire open reading frame (ORF) of the *lasB* gene-encoding pseudolysin was amplified from *P. aeruginosa* SWJSS3, and its detailed nucleic acid sequences (1497 bp) were confirmed by sequencing. Multiple sequence alignment with a *lasB* gene from the *P. aeruginosa* standard strain PA01 (Uniprot Entry: P14756) revealed 23 single-nucleotide mutations (corresponding to five amino acid mutations: Q102R, S241G, D244N, K282N, and R471S) in the *lasB* gene derived from *P. aeruginosa* SWJSS3 (Figure S1). These mutations in the lasB gene of *P. aeruginosa* SWJSS3 accumulated naturally through evolution. Researchers have examined the heterologous expression of pseudolysins, which were separated from *P. aeruginosa* in various environments, such as soil from natural sources [34], crude oil-contaminated soil [13], and feather dumping soil [9]. Interestingly, all these *P. aeruginosa* pseudolysins have the identical amino acid sequences of the mature domain, such as the pseudolysin from the *P. aeruginosa* standard strain, PA01. Therefore, in this study, we examined the mutant pseudolysin from *P. aeruginosa* SWJSS3 in *S. cerevisiae*.

### 3.2. Heterologous Expression of Pseudolysin by S. cerevisiae

We chose the *S. cerevisiae* strain B184M for pseudolysin expression. Strain B184M was obtained by UV mutagenesis and validated its superior protein production capacity by secreting α-amylase [30], human serum albumin [35], and affibody molecules [36,37]. Pseudolysin is a zinc metalloprotease with particular sites in the catalytic region for binding zinc and calcium ions [38]. The removal of cofactors from mature pseudolysin resulted in a complete loss of activity [39]. Pseudolysin expression might be restricted by the insufficiency of metal ions in the medium. Therefore, ZnCl_2_ and CaCl_2_ were additionally supplied in the YPD medium. The pseudolysin with the original ORF (strain B_lasB2) was employed to evaluate the addition of ZnCl_2_ and CaCl_2_ to enzyme activity and strain growth.

**Figure 1:**
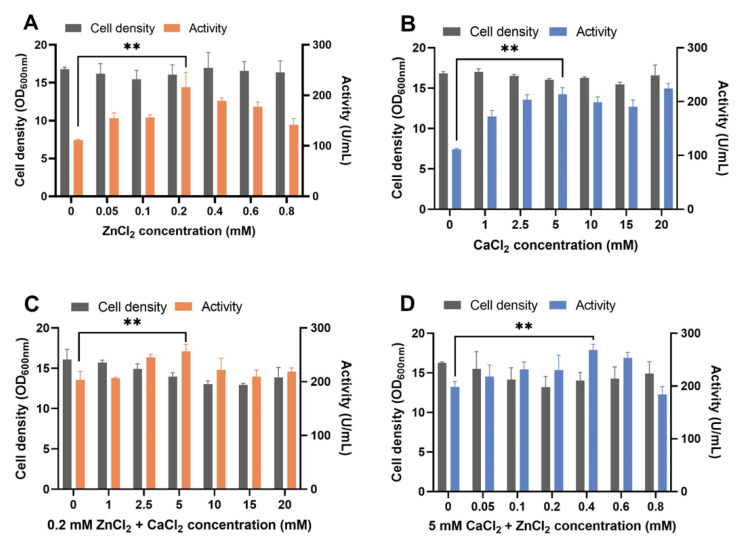
Recombinant pseudolysin expression optimization by adding ZnCl_2_ and CaCl_2_ to the YPD medium. (**A**) Effect of supplement with different concentrations of ZnCl_2_ (0–0.8 mM) in YPD medium on B_lasB2 strain cell density and proteolytic activity. (**B**) Effect of supplement at different concentrations of CaCl_2_ (0–20 mM) in YPD medium on B_lasB2 strain cell density and proteolytic activity. (**C**) Effect of supplement at 0.2 mM ZnCl_2_ and 0–20 mM CaCl_2_ in YPD medium on B_lasB2 strain cell density and proteolytic activity. (**D**) Effect of supplement with 5 mM CaCl_2_ and 0–0.8 mM ZnCl_2_ in YPD medium on B_lasB2 strain cell density and proteolytic activity. ** *p* < 0.01.

ZnCl_2_ or CaCl_2_ supplements in YPD medium increased proteolytic activity in the supernatant (Figure 1A,B). The highest proteolytic activity was reached when 0.2 mM ZnCl_2_ or 5 mM CaCl_2_ was added. The supplement of ZnCl_2_ and CaCl_2_ together showed additive effects (Figure 1C,D). The maximum activity was achieved in the YPD medium supplied with 0.4 mM ZnCl_2_ and 5 mM CaCl_2_ (Figure 1D) and was 2.41 times more than that of the YPD medium used as a control. When we supplied the Zn ion to the medium by using ZnSO_4_, which has SO_4_^2-^ as the anionic ligand of Zn^2+^, a similar result was observed (Figure S2). Regarding the heterologous expression of metal enzymes in *S. cerevisiae*, the metal ions were assembled into enzymes intracellularly and increased enzyme production [40]. Thus, in this work, increased protease activity by adding metal ions of ZnCl_2_ and CaCl_2_ may contribute to increased extracellular protease concentration. Besides serving as a cofactor for pseudolysin, Zn^2+^ is the cofactor of various other enzymes and proteins as well. Approximately 3% of proteins within a yeast cell contain zinc-binding domains [41], including alcohol dehydrogenase Adh1, Adh3, and Adh4 in the glycolytic pathway; superoxide dismutase Sod1; transcriptional factor zinc finger proteins; etc. [42]. Therefore, the supply of Zn and Ca may affect cellular metabolism. Adding metal ions slightly reduced the growth of yeast cells, especially in case of the supply of ZnCl_2_ and CaCl_2_ together (Figure 1C,D). Previous studies showed that adding exogenous zinc [43] or copper [44] decreases yeast cell growth. Excess metal ions generated reactive oxygen species and activated genes involved in the oxidative stress response [45,46]. Thus, cell growth stress caused by adding ZnCl_2_ and CaCl_2_ may be relieved by adjusting cellular metal ion homeostasis. For instance, vacuolar membrane transporters, such as Zrc1 [47] and Pmc1 [48], transport Zn^2+^ and Ca^2+^ from the cytosol to the vacuole for storage, which helps to increase the ionic stress resistance and reduce cytotoxicity.

### 3.3. Native Propeptide Is Essential for Pseudolysin Secretion in S. cerevisiae

**Figure 2:**
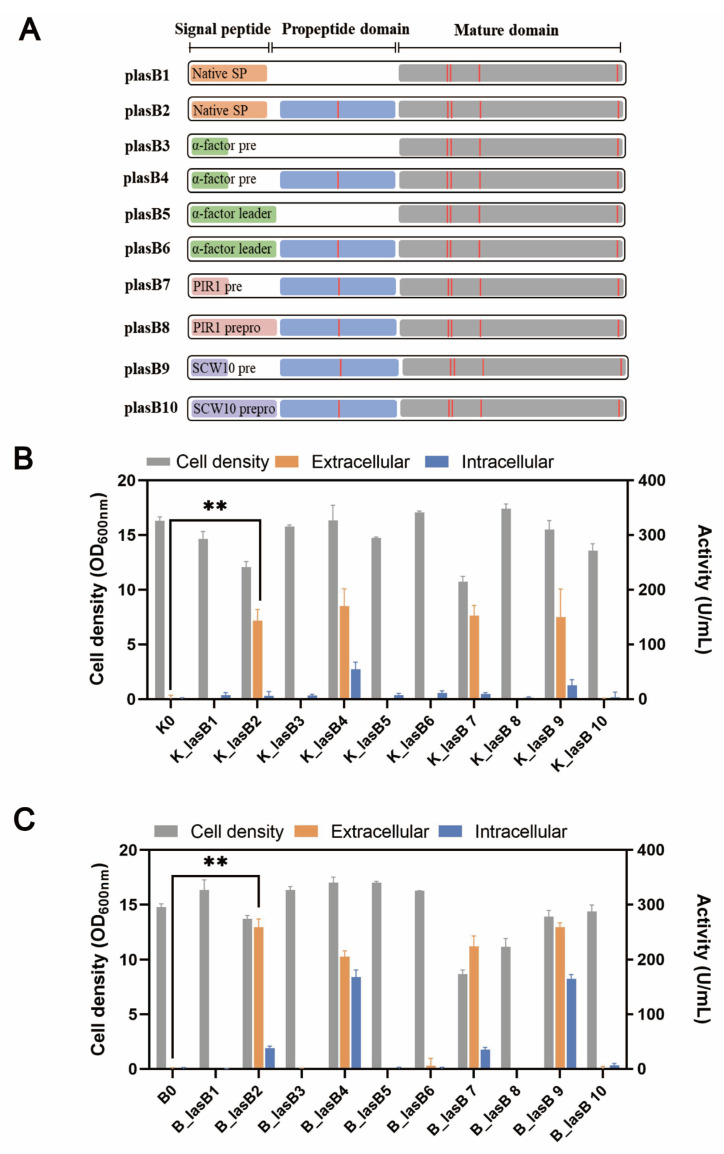
The protease production in CEN.PK 530-1C and B184M with different expression cassettes. (**A**) Pseudolysin expression cassette. Orange bar: native signal peptide of pseudolysin; short green bar: pre-region of α-factor leader; long green bar: complete α-factor leader; short grey pink bar: pre-region of PIR1 signal leader; long grey pink bar: complete PIR1 signal leader; short light purple bar: pre-region of SCW10 signal leader; long light purple bar: complete SCW10 signal leader; blue bar: propeptide of pseudolysin; gray bar: mature domain of pseudolysin; red vertical lines: five mutation sites found in mutant pseudolysin. (**B**) Cell density and proteolytic activity of *S. cerevisiae* strain CEN.PK 530-1C with different pseudolysin expression cassettes. (**C**) Cell density and proteolytic activity of *S. cerevisiae* strain B184M with different pseudolysin expression cassettes. ** *p* < 0.01.

Typically, a pseudolysin consists of a native signal peptide, a propeptide, and the mature domain. The propeptide, serving as a chaperone, is critical for the proper folding and secretion of pseudolysin [38]. Previously, Lin et al. showed that pseudolysin without its original propeptide region could not be secreted by *P. pastoris* [21]. We wondered whether the original propeptide region was essential for the recombinant expression of pseudolysin in *S. cerevisiae*. Therefore, we constructed different expression cassettes for examination (Figure 2A). Within these constructions, the α-factor leader was included, along with the signal peptide from pseudolysin. All the constructions were tested in two different yeast strains, the mutant yeast strain B184M and its parental strain CEN.PK 530.1C.

Consistent with the results of Lin et al. [21], only pseudolysin with its original propeptide was secreted by *S. cerevisiae* (Figure 2B,C). This indicated the decisive role of propeptide in producing mature pseudolysin. Interestingly, when using the entire length of the α-factor leader, whether the original propeptide region was present or not, the pseudolysin was not secreted. The α-factor leader is one of the most used signal peptides for protein secretion in eukaryotes. Various heterologous enzymes were successfully expressed both in *S. cerevisiae* and *P. pastoris* by using the α-factor leader, such as laccase [49], α-amylase [30], and protease [50,51]. The entire α-factor leader comprises a pre-region (19 amino acids), a pro-region (66 amino acids), and a STE13 cleavage site (EAEA tetrapeptide) [52]. The α-factor pro-region may remain attached to the cargo protein until it reaches the Golgi for cleavage by Kex2 [53]. The presence of an α-factor pro-region might hinder the original propeptide region of pseudolysin. We tested two other signal leaders from *S. cerevisiae*, PIR1 and SCW10, for pseudolysin secretion. The full length of PIR1 and SCW10 also consists of a pre-region and pro-region. Similarly, when using the full length of the PIR1 and SCW10 signal leaders for pseudolysin secretion, no enzymatic activity was detected. Once the pro-region was removed and we only used the pre-region as a secretion guider, the proteolytic activity increased (Figure 2B,C). This emphasized the importance of the original propeptide of pseudolysin. The lack of the propeptide of pseudolysin or an additional pro-region in front of the propeptide of pseudolysin hindered the maturity of pseudolysin. Compared with the parental strain CEN.PK 530-1C, the B184M strain had a better performance in the expression of pseudolysin. The highest extracellular proteolytic activity of pseudolysin in yeast strains with different expression cassettes was 258.95 U/mL (B_lasB2), which is comparable with another pseudolysin produced by *P. pastoris* [22].

### 3.4. Purification of Pseudolysin

**Figure 3:**
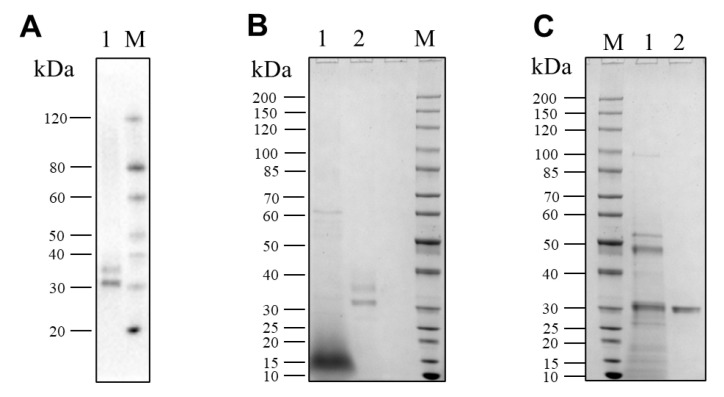
Western blot and SDS-PAGE analysis for SC-lasB and PA-lasB after protein purification. (**A**) Lane M: WB Protein Standard (M00521, Genscript). Lane 1 refers to the protein bands binding by the His-tag antibody from the fermentation supernatant of B_lasB2 strain. (**B**) Lane M: Protein Ladder (P0063, Beyotime). Lane 1: the fermentation supernatant of B_lasB2. Lane 2: the purified SC-lasB from B_lasB2. (**C**) Lane M: Protein Ladder (P0063, Beyotime). Lane 1: the fermentation supernatant of *P. aeruginosa* SWJSS3. Lane 2: the purified PA-lasB from *P. aeruginosa* SWJSS3.

**Figure 4:**
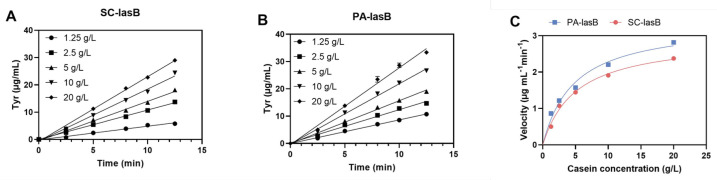
Estimation of the enzyme kinetic of SC-lasB and PA-lasB. (**A**) The amount of tyrosine released (μg/mL) during different reaction times for SC-lasB. (**B**) The amount of tyrosine released (μg/mL) during different reaction times for PA-lasB. (**C**) Michaelis–Menten plot of SC-lasB and PA-lasB at different concentrations of casein.

The recombinant pseudolysin secreted by *S. cerevisiae* (named SC-lasB) with a 6×His-tag was detected by Western blot assay, and two bands at 30–40 kDa were observed (Figure 3A). The recombinant pseudolysin was purified from the supernatant, followed by an SDS-PAGE analysis (Figure 3B), which agreed with the result of the Western blot assay. Band variants indicated that the recombinant pseudolysin had glycosylated modifications by yeast cells. We used the N-glycosylation site prediction tool NetNGlyc [54] to analyze the mutant pseudolysin and found N-linked glycosylation sites. Glycosylation on pseudolysin also occurred in the expression of *P. pastoris* [22]. The mutant pseudolysin produced by *P. aeruginosa* SWJSS3 (named PA-lasB) was purified, and only a single band was shown on the gel (Figure 3C). We measured the kinetic parameters of the two pseudolysins (Figure 4). The Kcat/Km value of SC-lasB was lower than that of PA-lasB when using casein as the substrate. This result suggested a decrease in the catalytic efficiency of recombinant pseudolysin from yeast cells compared with the mutant pseudolysin from *P. aeruginosa* SWJSS3 (Table 3).

### 3.5. Evaluation of Enzymatic Characteristics

**Figure 5:**
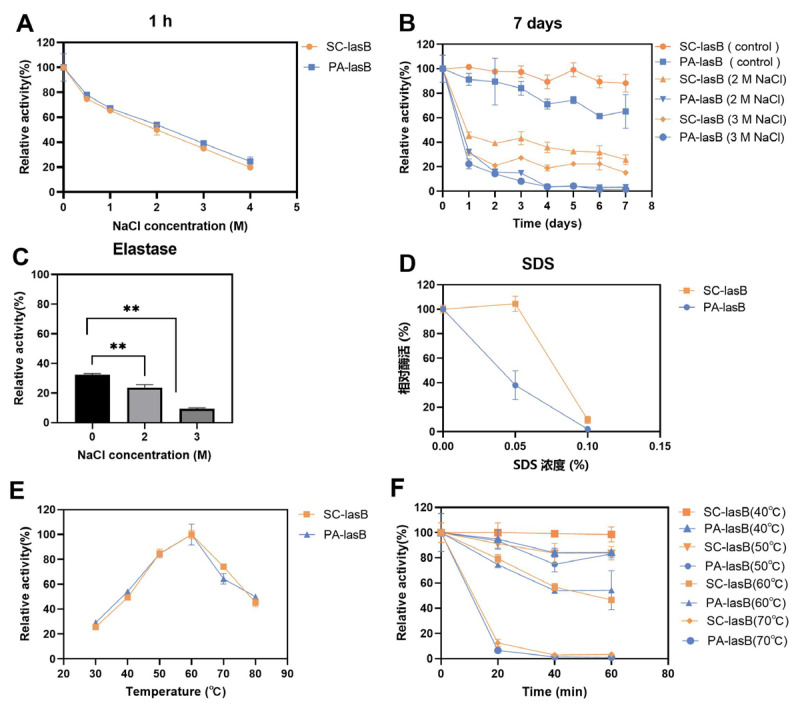
Assessment of the salt tolerance and thermal stability of SC-lasB and PA-lasB. (**A**) The relative activity after storage in 0–4 M NaCl for 1 h. (**B**) The relative activity after storage in 2 M and 3 M NaCl for 7 days. (**C**) The salt tolerance of elastase derived from porcine pancreas after storage in 2 M and 3 M NaCl for 7 days. (**D**) The relative activity after storage in 0–0.1% SDS for 1 h. (**E**) The optimal temperature curves of SC-lasB and PA-lasB. (**F**) The thermal stability of SC-lasB and PA-lasB. ** *p* < 0.01.

The purified pseudolysins SC-lasB and PA-lasB were evaluated for their salt tolerance and thermal stability. Although the increased concentration of NaCl led to decreased enzyme activity, both SC-lasB and PA-lasB retained at least 20% activity even in 4 M NaCl for 1 h (Figure 5A). Furthermore, we measured the residue activity of these two pseudolysins in a solution of NaCl for 7 days to reveal their long-term salt tolerance. The recombinant mutant pseudolysin SC-lasB showed 25.79% and 15.19% residue activity in 2 M and 3 M NaCl, respectively (Figure 5B). However, PA-lasB was almost completely inactivated after 7 days. The expression of pseudolysin in *P. pastoris* also resulted in higher protein stability than pseudolysin from *P. aeruginosa* [22]. N-glycosylation is a common post-translational modification in eukaryotic and helps protect the protein from degradation [55], reducing the aggregation tendency [56], and improving protein stabilization [57]. For recombinant pseudolysin, Han et al. [32] reported the mutation of any potential N-glycosylation site led to the decrease of solvent stability and thermostability. Thus, N-glycosylation was inferred to contribute to the enhanced salt tolerance of SC-lasB after its heterologous expression in *S. cerevisiae*. Moreover, pseudolysin is also known as elastase for its function in hydrolyzing elastin. We tested the salt tolerance of a commercial elastase derived from porcine pancreas. The commercial elastase retained only 9.41% in 3 M NaCl after 7 days (Figure 5C). Furthermore, a salt-tolerant alkaline protease secreted by a soy sauce fermentation strain *Aspergillus oryzae* 3.042 was also reported to retain over 20% relative activity after being stored in 3 M NaCl for 7 days [58]. The result indicated a potential application of recombinant pseudolysin in saline environments.

SDS, an ionic detergent, is usually used as a strong protein denaturant [59]. Adding SDS impaired pseudolysin activity. The residue activity of the pseudolysin PA-lasB was reduced by 62.02% at a concentration of 0.05% SDS compared with the untreated control group. In contrast, the recombinant pseudolysin SC-lasB was almost unaffected (Figure 5D). The SDS-induced protein denaturation mechanism depends on the SDS concentration [60]. When the concentration is below the critical micelle concentration (CMC) of SDS, which is 8.08 mM (0.23%) in water [61], SDS exists as a monomer and binds to protein residues by hydrophobic interaction and induces the unfolding of protein tertiary structure. Glycosides in secreted eukaryotic proteins might shield surface-exposed hydrophobic residues from solvent (Karpusas et al., 1997). We speculated that a resistance of the recombinant pseudolysin to SDS may also be beneficial for glycosylation, restricting SDS monomer access to hydrophobic residues. However, when the SDS concentration increased to 0.1%, both SC-lasB and PA-lasB were almost completely inactivated. This indicated the structure of SC-lasB and PA-lasB were severely impaired. The additional glycosides of SC-lasB could not provide protection anymore toward increased SDS monomer.

In addition to salt tolerance, the optimum temperature and thermal stability of pseudolysins were also measured. Interestingly, both pseudolysins had an optimum temperature of 60 °C (Figure 5E). This temperature was consistent with a native pseudolysin derived from *P. aeruginosa* MN7 [62] and *P. aeruginosa* C11 [9] and recombinant pseudolysin derived from *E. coli* JM109 [63,64]. Pseudolysins also had similar thermal stability of over 40–70 °C for 0–60 min (Figure 5F). It appeared that the optimal temperature and thermal stability of enzymes was not affected by the expression system.

### 3.6. Hydrolyzation of LD-SPI and HD-SPI in a High-Salt Environment

**Figure 6:**
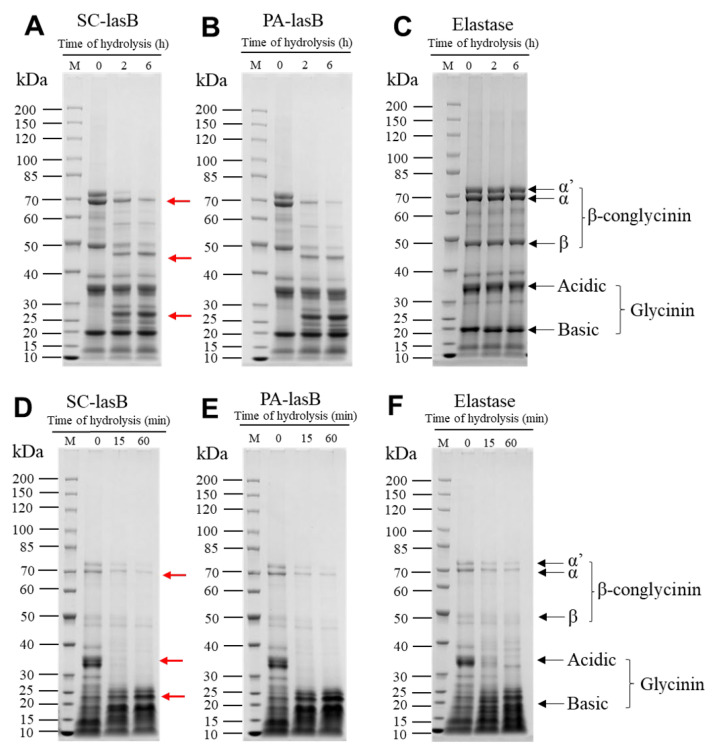
SDS–PAGE patterns of soy protein isolate hydrolysis obtained by SC-lasB and PA-lasB treatment (60 °C, pH = 7.0) with elastase from the porcine pancreas (50 °C, pH = 8.0) as the control group. (**A**–**C**) refer to the proteolysis of low-denatured soy protein isolate (LD-SPI). (**D**–**F**) refer to the proteolysis of high-denatured soy protein isolate (HD-SPI). Lane M: Protein Ladder (P0063, Beyotime). Red arrows represent protein bands that were degraded or newly generated.

Soy protein is a by-product of soybean oil processing and can be used as the primary raw material for soy product fermentation [65]. Thus, to evaluate the hydrolytic ability of pseudolysin in a high-salt environment, we dispersed soy protein isolate (SPI) into a 3 M NaCl solution and added two pseudolysins. LD-SPI retained more intact SPI subunits (Figure 6A–C). The α’, α, and β subunits of LD-SPI β-conglycinin were degraded by the two pseudolysins and generated a new low molecular weight protein group (25–30 KDa, 40–50 kDa). In contrast, there were no significant changes in glycinin. In the elastase group as a control, both β-conglycinin and glycinin were rarely hydrolyzed (Figure 6C). β-conglycinin (7S globulins, trimer) and glycinin (11S globulin, hexamers) are two major components in soybean and make up 70%–80% of total soy protein [66,67]. Apart from different conformations, glycinin has three to four times the amount of methionine and cysteine per protein molecule than β-conglycinin [68]. According to the MEROPS database, pseudolysin recognizes alanine, glycine, leucine, and phenylalanine as preferable cleavage sites [69]. This may explain the lower digestion of glycinin in LD-SPI by pseudolysin.

For another commercial SPI (HD-SPI), the soy protein had a higher degree of denaturation. The α’, α, and β subunits in HD-SPI were fewer than those of LD-SPI (Figure 6D–F). The α’ subunit of β-conglycinin was further hydrolyzed by the pseudolysins, and the glycinin acidic subunit was completely hydrolyzed within 15 min (Figure 6D–F). Thus, to promote hydrolyzing SPI by pseudolysin in a high-salt environment, SPI can be deeply denatured to destroy the subunit structure and release more cleavage sites or be combined with other protease hydrolysis specificities for glycinin. Meanwhile, the hydrolysates produced from soy protein isolate also deserve attention, as they have been reported to exhibit antioxidant properties [70,71].

## 4. Conclusions

In this study, the gene encoded a protease of *P. aeruginosa* SWJSS3, which was isolated from deep-sea mud, which was sequenced, and mutations were identified. This protease was recognized as a pseudolysin and then expressed in *S. cerevisiae*, and the proteolytic activity in the supernatant was significantly increased upon optimizing the cofactor concentration of the medium. The propeptide of pseudolysin was key to the secretion of mature pseudolysin. Meanwhile, the *S. cerevisiae* expression system enhanced the salt tolerance of pseudolysin in regard to NaCl and SDS. Recombinant pseudolysin was able to efficiently degrade the β-conglycinin of LD-SPI and the glycinin of HD-SPI in 3M NaCl solution at a high temperature, exhibiting viability for protein hydrolysis under high-salt conditions. Our future work will focus on improving the salt tolerance and catalytic activity of recombinant pseudolysin through protein engineering.

## Figures and Tables

**Table 3:** Enzyme kinetic parameters of SC-lasB and PA-lasB.

Enzyme	Concentration (mg/mL)	Activity (U/mL)	Specific Enzyme Activity (U/mg)	Vmax (μg•mL^−1^•min^−1^)	Km (g•L^−1^)	Kcat (min^−1^)	Kcat/Km (L•g^−1^•min^−1^)	R^2^
SC-lasB	0.097	996.49	10269.18	2.95	5.08	5.15	1.01	0.991 ^1^
PA-lasB	0.108	1563.16	14503.53	3.37	4.78	8.38	1.75	0.972 ^1^

^1^: The determination coefficient R^2^ indicated the goodness of fit of the Michaelis–Menten equation.

## Data Availability

All relevant data of this study are presented. Additional data will be provided upon request.

## Supplementary Materials

The following supporting information can be downloaded at: https://www.mdpi.com/article/10.3390/biom13010083/s1, Table S1: Primers used in this study; Figure S1: Sequence alignment for amino acid (**A**) and nucleotide (**B**) sequences between the wildtype lasB gene from P. aeruginosa standard strain PA01 (Uniprot Entry: P14756) and the mutant lasB gene from P. aeruginosa SWJSS3. Multiple sequence alignment was performed using the Clustal Omega web server and visualized by the ESpript 3 web server; Figure S2. Substituting ZnSO_4_ for ZnCl_2_ in the YPD medium. (**A**) Effect of supplement with different concentrations of ZnSO_4_ (0–1 mM) in YPD medium on B_lasB2 strain cell density and proteolytic activity. (**B**) Effect of supplement with different concentrations of CaCl_2_ (0–20 mM) in YPD medium on B_lasB2 strain cell density and proteolytic activity. (**C**) Effect of supplement with 0.1 mM ZnSO_4_ and 0–20 mM CaCl_2_ in YPD medium on B_lasB2 strain cell density and proteolytic activity. (**D**) Effect of supplement with 5 mM CaCl_2_ and 0–1 mM ZnSO_4_ in YPD medium on B_lasB2 strain cell density and proteolytic activity. * *p* < 0.05; ** *p* < 0.01.
