# Supplementary material for: Expression of a Salt-Tolerant Pseudolysin in Yeast for Efficient Protein Hydrolysis under High-Salt Conditions
Source: Biomolecules. 2022 Dec 30;13(1):83. doi: 10.3390/biom13010083 (PMC9855795; doi:10.3390/biom13010083)
Supplement: Supplementary file 1 [file biomolecules-13-00083-s001.zip › biomolecules-2025558-supplementary.pdf]

Supplementary Materials

Expression of a Salt-Tolerant Pseudolysin in Yeast for Efficient Protein Hydrolysis under High-Salt Conditions

Xiufang Liu [1, 2], Qian Lu [1, 3], Han Xiao [1, 2], Yunzi Feng [1, 2], Guowan Su [1, 2], Mouming Zhao [1, 2] and Mingtao Huang [1, 2, *]

[1] School of Food Science and Engineering, South China University of Technology, Guangzhou 510641, China

[2] Guangdong Food Green Processing and Nutrition Regulation Technologies Research Center, Guangzhou 510650, China

[3] Guangdong Haitian Innovation Tech Co., Ltd., Foshan 528000, China

* Corresponding: huangmt@scut.edu.cn

This file contains:

Figure S1-S2

Table S1

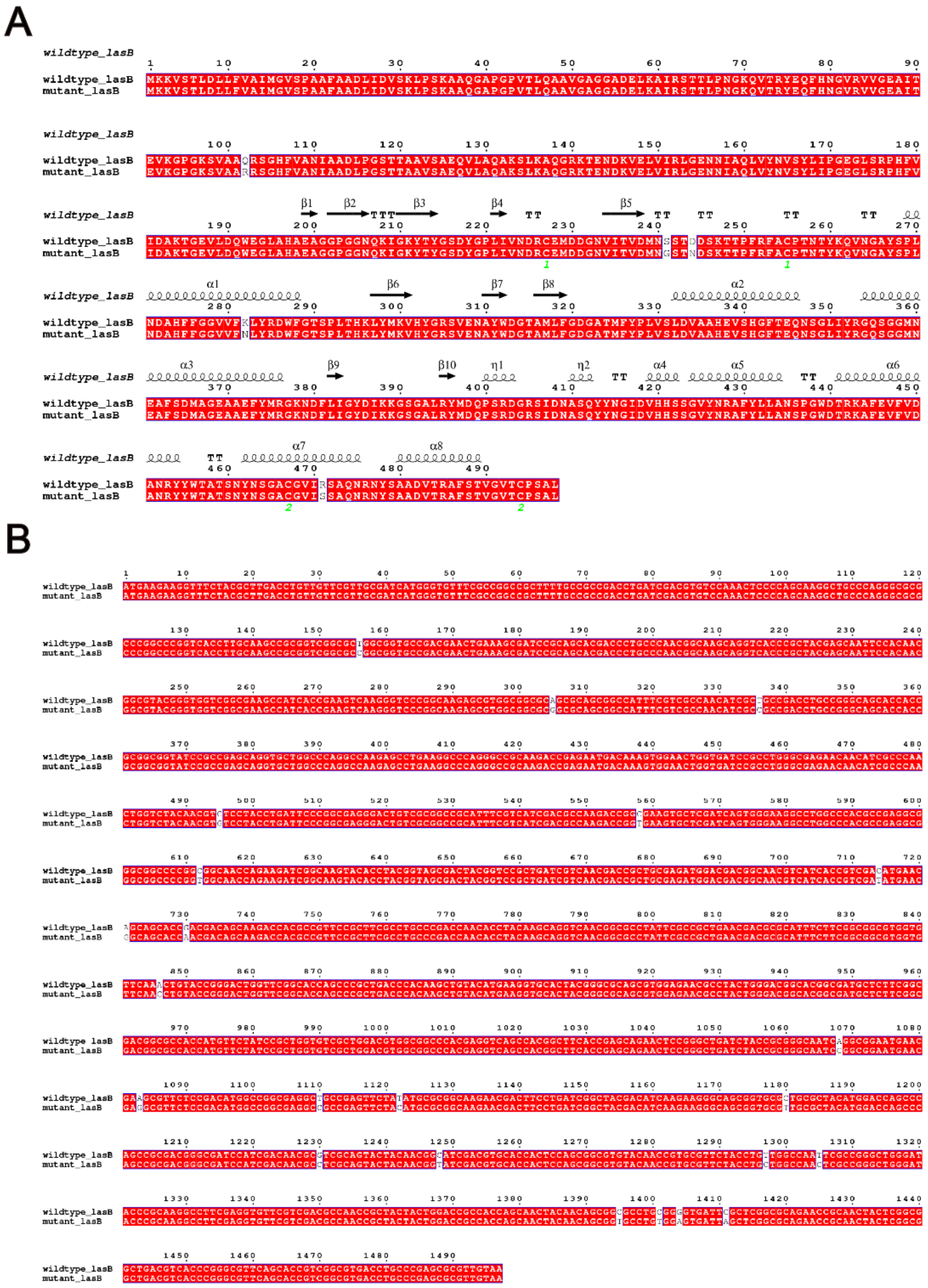

Figure S1. Sequence alignment for amino acid (A) and nucleotide (B) sequences between the wildtype *lasB* gene from *P. aeruginosa* standard strain PA01 (Uniprot Entry: P14756) and the mutant *lasB* gene from *P. aeruginosa* SWJSS3. Multiple sequence alignment was performed using the Clustal Omega web server and visualized by ESpript 3 web server.

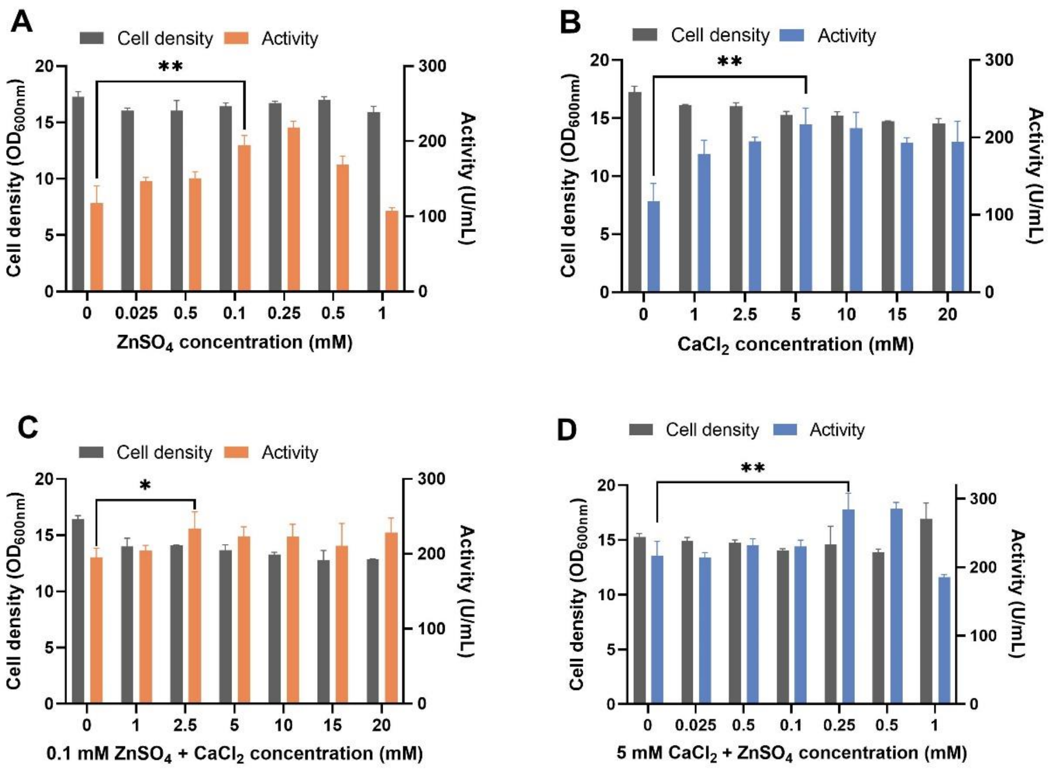

Figure S2. Substituting $ZnSO_4$ for $ZnCl_2$ in YPD medium. (A) Effect of supplement with different concentrations of $ZnSO_4$ (0-1 mM) in YPD medium on B_lasB2 strain cell density and proteolytic activity. (B) Effect of supplement with different concentrations of $CaCl_2$ (0-20 mM) in YPD medium on B_lasB2 strain cell density and proteolytic activity. (C) Effect of supplement with 0.1 mM $ZnSO_4$ and 0-20 mM $CaCl_2$ in YPD medium on B_lasB2 strain cell density and proteolytic activity. (D) Effect of supplement with 5 mM $CaCl_2$ and 0-1 mM $ZnSO_4$ in YPD medium on B_lasB2 strain cell density and proteolytic activity. * $p < 0.05$; ** $p < 0.01$.

Table S1. Primers used in this study

Primers	Description	Sequences
NPlasBF2	Obtain lasB gene from *P. aeruginosa* SWJSS3	CTAGGTACCATGAAGAAGGTTTCTACGCTTGACC
NPlasBR2		GAGGCTAGCTTACAACGCGCTCGGGCA
CPF	Primers for plasmid identification	AAAGGGCAGCATAATTTAGGAGTTTAG
lasBCYR		TACCGTCGTCCATTTCGCATC
lasBCYF		ACGCTAATCGGTATTATTGGACTG
CPR		TATCACTTGTAAATCTACCGTCCCT
AmpF		CTTTATCCGCCTCCATCCAGT
AmpR		TTTGCTCACCCAGAAACGC
NF	Plasmid plasB2 construction	CTAGGTACCAACAAAATGAAGAAAGTCTCTAC
lasBR		GAGGCTAGCTCAATGGTGATGATGGTG
NR1	Plasmid plasB1 construction with NF+lasBR	ACCACCGGCTTCAGCAGCAAACGCAGCTGGA
lasBF1		CCAGCTGCGTTTGCTGCTGAAGCCGGTGGTCC
AF	Plasmid plasB3 construction with lasBR	CTAGGTACCAACAAAATGAGATTTCCATCTATT
AR2		ACCACCGGCTTCAGCAGCCAAAGCAGAAGAAGCA
lasBF2		TCTTCTGCTTTGGCTGCTGAAGCCGGTGGTCC
AR3	Plasmid plasB4 construction with AF+lasBR	ATCAATCAAATCAGCAGCCAAAGCAGAAGAAGCA
lasbF3		TCTTCTGCTTTGGCTGCTGATTTGATTGATGTTTCC
AR4	Plasmid plasB5 construction with AF+lasBR	GCTTCAGCAGCTTCAGCCTCTCTTTTATCCAAAGAAACACCTTCTTCT
lasBF4		ATAAAAGAGAGGCTGAAGCTGCTGAAGCCGGTGGTCC
AR5	Plasmid plasB6 construction with AF+lasBR	AAATCAGCAGCTTCAGCCTCTCTTTTATCCAAAGAAACACCTTCTTCT
lasBF5		ATAAAAGAGAGGCTGAAGCTGCTGATTTGATTGATGTTTCC
PIR1_F	Plasmid plasB7 construction with lasBR	CTAGGTACCAACAAAATGCAATACAAAAAATCATTAGTTGCCT
PIR1_5		ATCAATCAAATCAGCAGCTAAAGATGTAGCAACTAAGGCG
PIR1_6		GCTACATCTTTAGCTGCTGATTTGATTGATGTTTCCAAGTTG
PIR1_3	Plasmid plasB8 construction with PIR1_F+lasBR	ATCAATCAAATCAGCTCTCTTAGCCTTGGAGGAAGCA
PIR1_4		TCCAAGGCTAAGAGAGCTGATTTGATTGATGTTTCCAAGTTG
SCW_F	Plasmid plasB9 construction with lasBR	CTAGGTACCAACAAAATGCGTTTTTCAAATTTCCTAACTGTATCT
SCW10_5		ATCAATCAAATCAGCACCTAGAGCTCCGGTTAATAATGC
SCW10_6		ACCGGAGCTCTAGGTGCTGATTTGATTGATGTTTCCAAGTTG
SCW10_3	Plasmid plasB10 construction with SCW_F+lasBR	ATCAATCAAATCAGCACGCTTTTCATGTTTATGGCGAACAGCAGGAGC
SCW10_4		AAACATGAAAAGCGTGCTGATTTGATTGATGTTTCCAAGTTG
